# A Center-Tapped Transformer Based Multifunctional Single-Phase Converter with Wide DC-Bus Control

**DOI:** 10.3390/s23042227

**Published:** 2023-02-16

**Authors:** Arinze Stephen Obi, Si-Heon Lee, Hyun-Sam Jung, Jae-Jung Jung

**Affiliations:** 1 Department of Electronic and Electrical Engineering, Kyungpook National University, Bukgu, Daegu 41566, Republic of Korea; 2 Department of Electronics and Electrical Engineering, Dongguk University, Seoul 04620, Republic of Korea

**Keywords:** AC-DC converter, center-tapped transformer, inverter, single-phase converter, wide DC-bus control, battery energy storage system

## Abstract

Alongside the rapid increase in distributed power generation and load, the demand for highly efficient and reliable power converters is increasing. This has resulted in the rise of grid interfaced renewable energy sources (RES), rapid deployment of battery energy storage systems (BESS) coupled with energy managment systems (EMS), and DC based grid. This paper presents a center-tapped transformer-based single-stage single-phase full-bridge (FB) bidirectional AC-DC converter and its control strategy to improve controllability and reliability in applications such as DC distribution, PV/BESS grid interfacing, vehicle to grid (V2G), and so on. In contrast to conventional galvanically isolated topologies, a single-phase center-tapped transformer is introduced. It links and galvanically isolates the converters and the grid and provides its leakage inductance as the needed inductor required for current control (depending on the design). Furthermore, it reduces the number of conventionally required power conversion stages by employing a wide DC-bus voltage control strategy, resulting in a single converter that undergoes a single power conversion. Additionally, the voltage level can be increased to further enhance the output quality by cascading multiple converters (Multi-Level). The structure, operation, and basic control scheme are discussed in detail. Verification through a 220 $V_{rms}$, 1.8 kVA, and 45∼100 $V_{DC}$ simulation and small-scale experimental prototype (60∼100 $V_{DC}$ voltage) for practical validation of the topology is also presented.

## 1. Introduction

The global drive for electrification and decarbonized energy generation has led to several developments in all levels of power generation, especially in extra-low-voltage and meduim-level-voltage distributed generation (DG) with diverse renewable energy sources [1,2,3,4] coupled with energy management systems (EMS) and DC grid applications. Likewise, the demand for bi-directional power flow has led to several novel converters categorized as both the line-commutated current source converter (CSC) and the self-commutating voltage source converter (VSC) topologies. Unlike CSC topologies, the VSC topology is preferred because it provides means for decoupled control of the reactive and active power components as well as requiring a smaller converter footprint. Large-scale operation is also now possible, thanks to advancements in high-voltage direct-current (HVDC) and multi-level topologies. These characteristics has led to several applications of VSC topologies in grid and renewables integration [5,6,7]. However, the variable outputs of renewable sources serving as the converter input DC source destabilizes a stable DC-bus voltage. This has lead to the inclusion of additional voltage stabilizing circuit in power conversion conversion stages.

**Figure 1:**
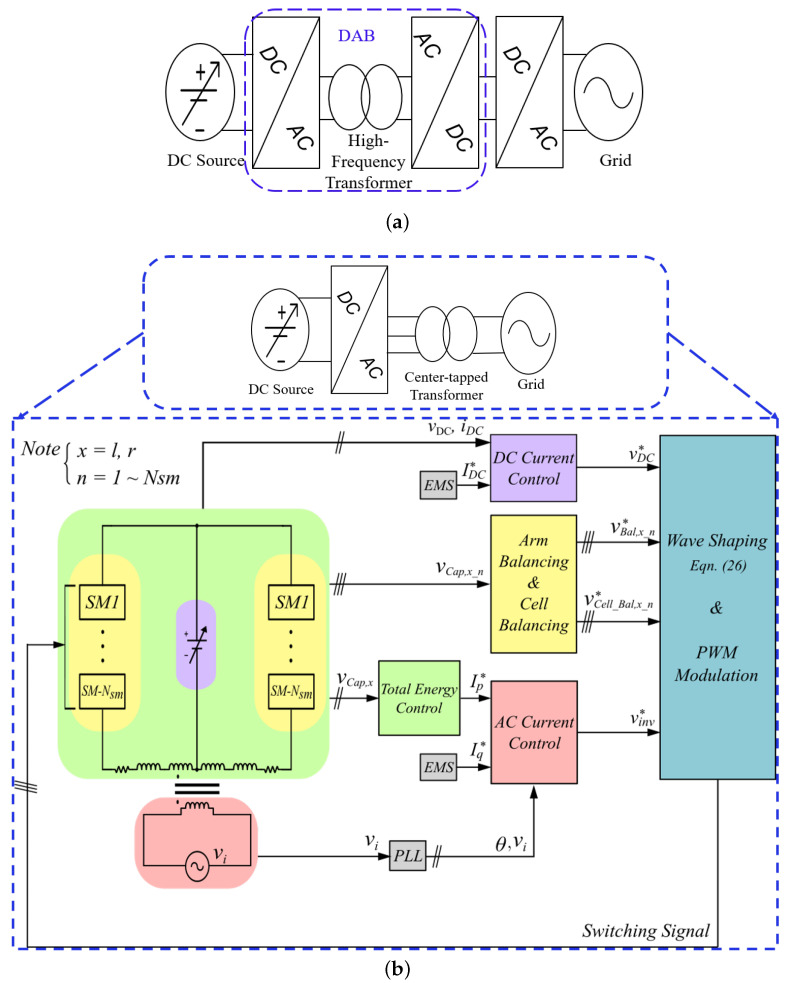

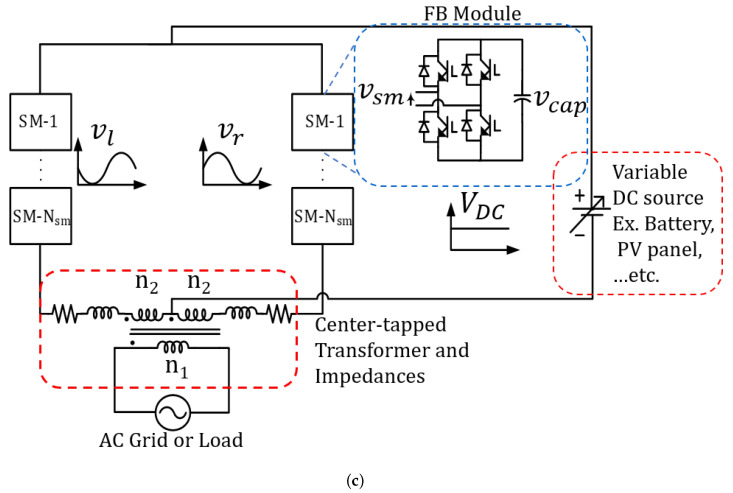
A generalized representation of the conventional topology (**a**), proposed single-stage topology (**b**), and circuit schematic of proposed topology (**c**).

As a result of local grid codes usually requiring isolation for grid interfaced converters, the integration of household renewables and storage batteries to the grid has increased interest in galvanically isolated power converters [8,9]. This added isolation provides enhanced safety by isolating the high voltage and low voltage sides and prevents DC offsets from flowing into the grid while also increasing power density. Such application of isolated topologies usually involves two stages or more with a high or low-frequency transformer, depending on if the isolation is on the DC or AC-side, respectively. For proper operation in the case of power being sent to the grid, stable input power is needed [10]. To achieve this, for example, the input source is boosted then converted to AC, stepped up via a transformer, and then several other AC-DC-AC conversions to meet required standards. Likewise, when power is being captured from the grid, similar steps are taken in reverse. In summary, many applications requires; a wide DC-bus range, low grid current distortion, high power factor and efficiency. A well-adapted approach is the dual active bridge (DAB) coupled with an inverter [11], as shown in Figure 1a. It involves two stages or more with a high-frequency isolation transformer, resulting in a small form factor.

This paper presents a center-tapped transformer-based bidirectional single-stage AC-DC multi-level converter (MLC) for wide DC-bus applications. A three-phase configuration of the converter with half-bridge SMs connected in parallel for MVL application was first introduced in [12]. Similarly, a series connected three-phase configuration was recently proposed in [13] for HVDC. In this paper, the proposed configuration employs a center-tapped transformer that links FB converters (capable of bidirectional power flow) in an arm structure to the AC grid. This results in a single multi-functional converter with a single power conversion stage with the modularity of modular multi-level converter (MMC) and capable of variable DC-side (source side) output control. This variability of the DC-side control makes it possible to achieve maximum power point tracking (MPPT) without an additional MPPT boost-stage in PV and grid interfacing application. Due to the equal power sharing of the arms, higher power ratings can be achieved. Furthermore, in the case of a DC short circuit (SC) fault, unlike in [12,13], the FB modules makes it capable of adequate DC FRT (fault ride-through). Additionally, The power quality can further be improved by cascading multiple sub-modules, resulting in better (voltage and current) total-harmonic distortion (THD) and low EMI [14]. This article presents:

- A detailed operational principle of the proposed configuration;
- Its comprehensive control strategies;
- Simulation and small-scale prototype validation.

The rest of this article is as follows: Section 2 describes the operation and mathematical derivation of equivalent circuits as well as voltage forming and circulating current considerations of the converter. Section 3 details the control strategy, and in Section 4 and Section 5, the simulation and experimental studies are described and the results discussed, respectively. Section 6 contains a conclusion.

## 2. Proposed Topology and Its Equivalent Circuit

This section presents the proposed converter topology, its equivalent circuit, and the principle of operation. In this study, it is assumed that the system is lossless, ripple-free and is in steady state.

### 2.1. Circuit Configuration

The generalized structure of the proposed converter is as depicted in Figure 1b; it involves only a single-stage power conversion and is connected to the grid via the transformer. It forgoes the in-between stages and the high-frequency transformer for a low-frequency center-tapped transformer. Although it may seem like a disadvantage, while providing required voltage isolation, it enables the decoupled control of both AC and DC components and also provides part of the needed inductance for grid interfacing and control implementation, which further reduces total system size. The system circuit schematic and steady-state waveforms is depicted in Figure 1b. As shown in Figure 1c, it comprises of *N_sm_* FB sub-modules (*SM-1, SM-2,⋯SM-N_sm_*) with cell capacitors in an arm structure for voltage forming, a variable DC voltage source, an AC source and a center-tapped transformer with turns $n_{1}\;\mathrm{and}\;n_{2}$ ($n_{1}$ and $n_{2}$ are grid and the converter-side respective number of turns). Here $v_{sm}$ is the inserted voltage by a particular sub-module.

**Figure 2:**
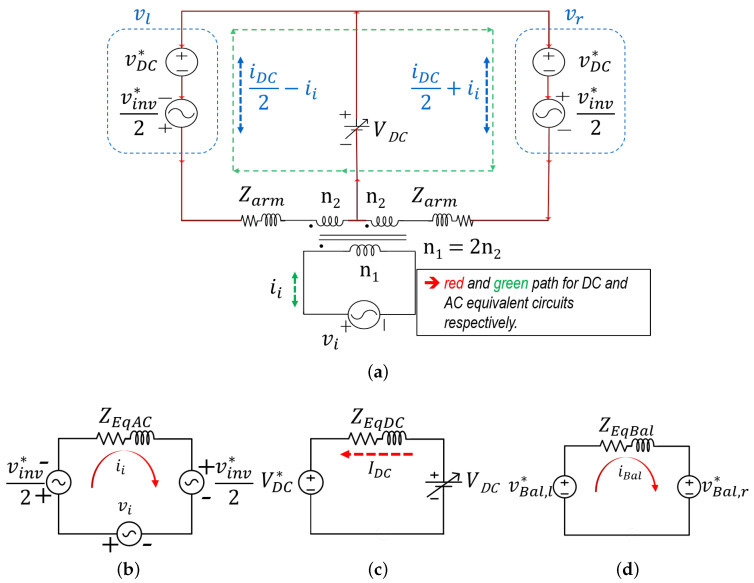
System equivalent circuit (**a**), AC equivalent circuit (**b**), DC equivalent circuit (**c**), and arm balancing equivalent circuit (**d**).

As shown in the equivalent converter circuit of Figure 2a, both DC and AC components exist in the converter arms. Ideally, each arm generates equal DC and equal AC voltages, with the AC components being equal proportions of the grid voltage (depending on the turn ratio) and is $180^{0}$ out of phase from each other. These components enters the transformer from opposite sides and in accordance with the dot convention, the DC components in the arm canceling at the transformer side, while the AC component is summed and transformed via transformer action. Conversely, the AC component cancels at the DC-bus, while the DC component appears in parallel. This characteristic enables the decoupled control of both DC and AC components, making it possible for a wide range of DC-bus voltage control without transformer core saturation. In this article, the analysis is based on such an ideal case. Ignoring the ripple components, losses, and assuming the converter switches are ideal, $n_{1}=2,\;n_{2}=1$ and the grid components are represented as:

(1) $$\left\{\begin{array}{cc} v_{i} & =V_{i}sin\left(wt\right)\\ i_{i} & =I_{i}sin\left(wt+\varphi \right). \end{array}\right.$$

where $I_{i},\;\mathrm{and}\;V_{i}$ are the peak of the grid current and voltage, respectively, and φ, the power angle. In steady state, the converter references (asterisked) can be assumed to be same as the output. Hence, with balanced arms, each arm produces full DC voltage ($V_{DC}^{*}$) and half the AC voltage ($v_{inv}^{*}$). Thus, the converter output to a reference as depicted in Figure 2a can be represented as:

(2) $$\left\{\begin{array}{cc} v_{l} & =V_{DC}^{*}-\frac{v_{inv}^{*}}{2}\\ v_{r} & =V_{DC}^{*}+\frac{v_{inv}^{*}}{2}. \end{array}\right.$$

where $v_{inv}$ is the voltage across the secondary of the transformer. Equation (2) is the converter inserted voltage as a result of pulsed width modulation (PWM) action to a given reference voltage. The current flowing in the arms as a result of the inserted voltage can be expressed as:

(3) $$\left\{\begin{array}{cc} i_{l} & =\frac{I_{DC}}{2}+i_{i}\\ i_{r} & =\frac{I_{DC}}{2}-i_{i}. \end{array}\right.$$

where the grid current can be accurately expressed in terms of the converter arm components as:

(4) $$i_{i}=\frac{i_{r}-i_{l}}{2}.$$

The green loop of the equivalent circuit in Figure 2a shows the AC current flow and can be represented as in (5). Rearranging the terms results in (6), where $Z_{EqAC}=2Z_{arm}$ is the AC equivalent impedance. Figure 2b is a pictorial representation of the AC equivalent circuit.

(5) $$\begin{array}{c} v_{inv}^{*}+Z_{EqAC}i_{i}-v_{i}=0. \end{array}$$

(6) $$v_{inv}^{*}+Z_{EqAC}i_{i}=v_{i}.$$

Conversely, the red loop shows the DC current path. The equivalent relationship seen from the DC-side is as in (7) and is graphically depicted in Figure 2c. Rearranging the terms results in (8). In contrast to the AC equivalent circuit, the DC equivalent impedance is $Z_{EqDC}=Z_{arm}/2$ and servers as the DC control plant.

(7) $$V_{DC}-V_{DC}^{*}-Z_{EqDC}I_{DC}=0.$$

(8) $$V_{DC}^{*}+Z_{EqDC}I_{DC}=V_{DC}.$$

However, it is worth noting that in the case of unbalanced arms, the circulating DC would induce a $2Z_{arm}I_{DC}$ term in (6) and the the AC, a $Z_{arm}i_{i}/2$ term in (8).

From the relationship in (6) and (8), it is clear that ideally, the DC components do not appear at the AC side and vice versa. Hence, the DC side requires little or no filter. However, the switching devices and transformer secondary side needs to be rated for:

(9) $$I_{arm\_rms}=\sqrt{{\left(\frac{I_{DC}}{2}\right)}^{2}+\frac{I_{i}^{2}}{2}}.$$

### 2.2. Arms’ Power Balance and Circulating Current Considerations

#### 2.2.1. Arms’ Power Balance

Considering the left and right arms of the converter, the instantaneous power carried by each can be expressed as:

(10) $$p_{l,r}=v_{l,r}i_{l,r}$$

Substituting (2) and (3) into (10) results in:

(11) $$\begin{array}{cc} p_{l,r} & =\frac{V_{DC}^{*}I_{DC}}{2}-\frac{V_{inv}^{*}I_{i}cos\left(\varphi \right)}{4}+V_{DC}^{*}I_{i}sin\left(wt+\varphi \right)\\ \; & -\frac{V_{inv}^{*}I_{DC}sin\left(wt\right)}{4}+\frac{V_{inv}^{*}I_{i}cos\left(2wt+\varphi \right)}{4} \end{array}$$

Averaging the above outcome of (11) over the grid period results in the average active power as expressed below:

(12) $$<p_{l,r}>=\frac{V_{DC}^{*}I_{DC}}{2}-\frac{V_{inv}^{*}I_{i}cos\left(\varphi \right)}{4}.$$

From the above result of (12) it is clear that each arm carries half of the total rated power. Considering that in steady-state, the average power contributed by each arm is zero results in:

(13) $$\begin{array}{cc} \; & \frac{V_{DC}^{*}I_{DC}}{2}=\frac{V_{inv}^{*}I_{i}cos\left(\varphi \right)}{4}\\ \; & I_{DC}=\frac{V_{inv}^{*}I_{i}cos\left(\varphi \right)}{2V_{DC}^{*}}. \end{array}$$

This can be further simplified in terms of apparent power *S* as:

(14) $$I_{DC}=\frac{Scos(\varphi )}{V_{DC}^{*}}=mI_{i}cos\left(\varphi \right).$$

where m(0≤m) is the ratio of the peak AC to DC bus voltage in an arm and is defined as:

(15) $$m=\frac{V_{inv}^{*}}{2V_{DC}^{*}}.$$

#### 2.2.2. Circulating Current Consideration

The main concern here is the possibility of asymmetry between arms, as even a little DC mismatch can result in DC flux that may cause transformer saturation [15]. However, this can be mitigated by implementing a sort of fail-safe in the AC controller to ensure mitigation of circulating DC current. The effect of circulating current due to ripple in the arm voltage is an area of great importance. A comprehensive study in the case of conventional MMC has been carried out in [16]. Although not mathematically included here, the effect of circulating ripple current is quite different in the proposed converter as discussed in [13]. Due to the arm voltages being equal opposites, the dominant twice-the-line-frequency ripple present in MMC cancels in this converter. This is the same for all even ripple components. Conversely, the odd ripple components add and result in circulating current. However, this is of negligible quantity.

### 2.3. Cell Capacitor Sizing

The selection of the cell capacitor is a very important design aspect because it can be said to be the bedrock of the converter. It relates the maximum allowable peak voltage ripple ΔV to the required minimum capacitance $C_{sm}^{min}$. From [17], the minimum required capacitance can be represented in terms of the peak to peak arm energy deviation ΔE over a fundamental period and the allowable peak voltage ripple as:

(16) $$C_{sm}^{min}=\frac{\Delta E_{arm}}{2N_{sm}{\left(V_{cap}^{nom}\right)}^{2}\Delta V}.$$

where $N_{sm}$ the required sub-module per arm can be expressed as:

(17) $$N_{sm}=\frac{V_{DC}\left(1+m\right)}{V_{cap}^{nom}}.$$

From the instantaneous power in the arm (10), the instantaneous energy can be determined as:

(18) $$e_{l,r}=\int p_{l,r}dt.$$

Substituting (14) and (15) into (18) results in the instantaneous energy deviation per arm:

(19) $$\begin{array}{cc} e_{l,r}=\frac{s}{4mw}(m^{2}cos\left(wt-\varphi \right) & +\left(m^{2}-2\right)cos\left(wt+\varphi \right)\\ \; & +msin(2wt+\varphi )). \end{array}$$

Since converter sizing is preferably considered in energy storage per apparent power (VA), (19) can be rearranged independent of VA rating and frequency as:

(20) $$\begin{array}{cc} \frac{w\cdot e_{l,r}}{S}=\frac{1}{4m}(m^{2}cos\left(wt-\varphi \right) & +\left(m^{2}-2\right)cos\left(wt+\varphi \right)\\ \; & +msin(2wt+\varphi )). \end{array}$$

Thus, from (20) the minimum required capacitance in (16) can be rewritten as:

(21) $$C_{sm}^{min}=\frac{S\Delta E_{arm}}{2wN_{sm}{\left(V_{cap}^{nom}\right)}^{2}\Delta V}.$$

**Figure 3:**
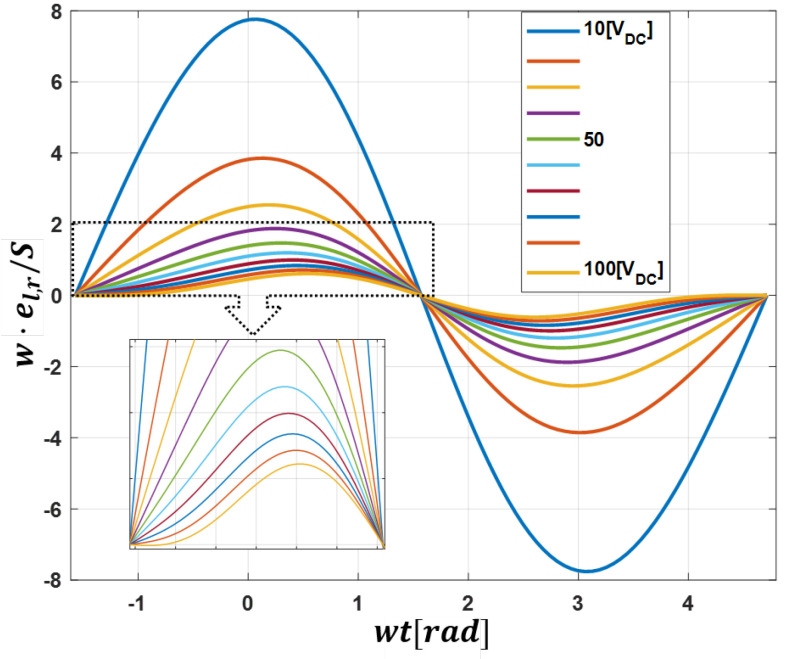
Converter energy per VA plot for varying levels of DC voltage (stepsof10 V) and a constant grid voltage of 220 $V_{rms}$.

Figure 3 shows the converter arm energy deviation over a fundamental period for different values of *m* (varying DC voltage). In this way, the peak-to-peak energy deviation required for a particular voltage can be obtained. This can be substituted in (20) for determination of minimum required capacitor value. Additionally, other factors such as capacitor ripple current rating, lifetime, etc., determine the final configuration. In applications requiring wide DC-bus operation, it is good practice to design for the required minimum DC voltage level.

## 3. Control Strategy

The general control objectives of the proposed topology are very similar to already existing grid-connected VSC topologies, which usually is an energy controller acting on the active power component of the AC-side controller [18,19]. Ideally, in the proposed topology, the components of each arm are precisely equal in magnitude, and hence, like the components, cancel at respective points. However, in practical application, as that the system is built on FB modules that use a finite energy source capacitor and semiconductor switches for operation, it is expected for there to be a slight arm impedance mismatch. This can lead to drastic energy differences between capacitors and the DC flowing into the transformer; this may cause it to be saturated. Thus, the system (capacitors) total energy control, arm energy balance control and a control ensuring the DC components sum to zero are needed. Lastly, a DC-side controller can also be implemented to obtain desired DC-side current.

Depending on the application, the total energy control can be achieved from either side (DC or AC); for demonstration purpose, the grid-side energy control will be discussed. Therefore, in a two-loop control fashion, a much slower total energy controller ensures energy balance between the DC and AC sides by acting on the active current component of the AC via a much faster AC controller (opposed to DC controller in the case of DC-side total energy control). In this way, maintaining the energy in the converter at a sustainable reference ensures proper operation is achieved. The additional controller ensures that energy sharing between both arms is balanced by forcing the arm with more energy to generate slightly more power and the other less. The DC-side controller (power dispatcher) produces the required converter voltage offset depending on the DC error term. Each controller is further discussed below.

### 3.1. Total Energy and AC Current controller

**Figure 4:**
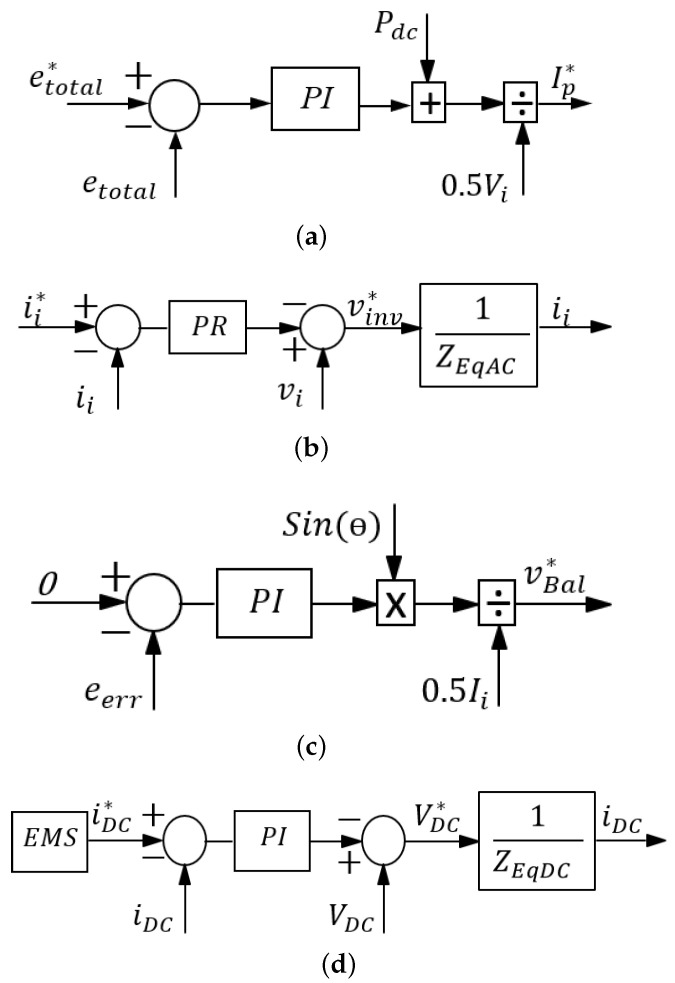
Total energy controller (**a**), AC controller (**b**), arm balancing controller (**c**), DC controller (**d**).

As aforementioned, the system’s total energy controller ensures the input energy satisfies the requirements of the DC-bus. Assuming equal energy injection into the capacitors and ignoring voltage ripple component, the system total energy relationship can be described as:

(22) $$\begin{array}{cc} e_{total} & =\frac{1}{2}C_{sm}\sum_{n=1}^{Nsm}V_{cap,l\_n}^{2}+\frac{1}{2}C_{sm}\sum_{n=1}^{Nsm}V_{cap,r\_n}^{2}\\ \; & =e_{l}+e_{r}. \end{array}$$

where $e_{l}\;\mathrm{and}\;e_{r}$ are the energy stored in the left and right arm capacitors, and $e_{total}$ the sum total of both. For control purposes, the energy captured by the DC bus $\int P_{dc}dt$ is considered a disturbance and is compensated for through feedforward $P_{dc}$. Thus, the total energy control plant can be derived as:

(23) $$\begin{array}{cc} \frac{de_{total}}{dt} & =\frac{V_{i}\cdot I_{p}^{*}}{2}=P_{ac}\\ \frac{e_{total}\left(s\right)}{p_{ac}\left(s\right)} & =\frac{1}{s}=G_{p}\left(s\right). \end{array}$$

where $I_{p}^{*}$ is the active current component of the AC current reference $i_{i}^{*}$. A proportional-integral (PI) controller tuned based on (23) and considering the inner loop dynamics acts on the total energy error, and sets the active power reference as depicted in Figure 4a.

For the AC control, the grid-side current or the arm currents can be sampled, summed as in (4), and used instead for current control. The latter is recommended because not only does it decrease the number of required sensors, any difference in the arms DC components appears as an offset in the summation. The controller, in turn, produces an output with DC correctional terms as well. In a well designed system, this term is minimal in that it does not affect the central current control. In this way, any DC component mismatch is automatically resolved, even during transients.

A single phase-locked loop (PLL) based on a standard quadrature signal generator (QSG) is used to synchronize the grid and the converter and also obtain the grid side voltage magnitude [20,21]. The second order generalized integrator (SOGI) PLL is utilized for its resilience to grid voltage harmonics. If θ is the extracted grid phase angle, then converter AC reference current $i_{i}^{*}$ can be decomposed into active and reactive components, respectively, as $I_{p}^{*}$ and $I_{q}^{*}$. This is represented in stationary frame as:

(24) $$i_{i}^{*}=I_{p}^{*}sin\left(\theta \right)+I_{q}^{*}cos\left(\theta \right).$$

A Proportional-Resonant controller (PR) is employed in the current control for its error-free tracking of sinusoidal reference in steady state and superior transient response compared to the PI controller [22,23,24]. From (6), the control block can be represented as in Figure 4b.

### 3.2. Arm Energy Balancing and DC Current Control

As aforementioned, discrepancies in arm impedance can cause energy imbalance, leading to unequal DC and/or AC components in each arm and possible system destabilization. Thus, it is paramount to ensure almost equal energy transfer from both arms. This can be realized by utilizing the circulating AC term to drive the difference between both arm energies to zero. This is implemented by generating an AC voltage correction term that adds to both modulation signals (since the AC terms are natural opposites) [13] or by generating a DC correctional term that adds to one arm and subtracts from the other. This introduces a common-mode voltage term (as in Figure 2d) that is very small compared to the converter voltage. Hence, aside from balancing both arm energies, it does not affect the overall system operation. The impedance $Z_{EqBal}$ is for illustration only. The control block for AC term-based correction is as depicted in Figure 4c. The PI controller works on the difference between the left and right arm capacitor energies ($e_{l}-e_{r}=e_{err}$), and generates a mean AC power term. This is converted to the balancing voltage term $v_{Bal}^{*}$, while taking into account the phase of $i_{i}$. The drawback of AC-based balancing is that, although small in magnitude, it introduces a sinusoidal term in the DC current. Furthermore, although not depicted, it is good practice to incorporate an anti-windup scheme in this control [25].

For the power dispatcher, a current reference is compared with the converter output DC for DC-side control, resulting in the required converter DC offset voltage as output. Assuming $V_{DC}$ in (8) is considered as disturbance and compensated for via feedforward, the control plant can be approximated as:

(25) $$\frac{I_{DC}\left(s\right)}{V_{DC}^{*}\left(s\right)}=\frac{1}{Z_{EqDC}}=G_{DC}\left(s\right).$$

A simple PI controller can be used for compensation. The control block diagram is as shown in Figure 4d. In the case of a converter DC-side SC fault, FRT is achieved by simply switching the DC reference current to zero. All controllers were designed for digital implementation with the fundamental knowledge as in [26].

**Figure 5:**
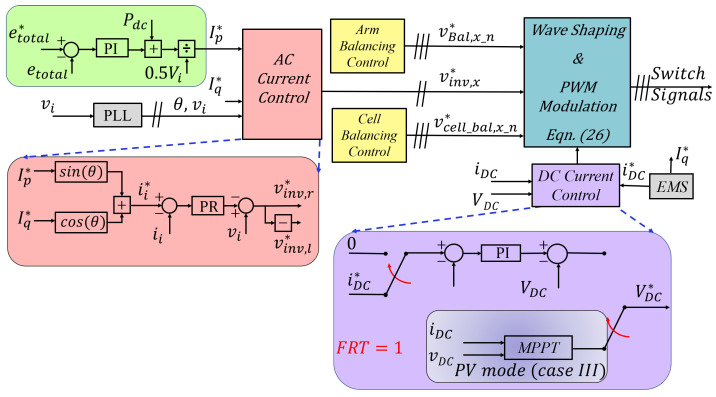
General control strategy for proposed converter.

For modulation, similar methods applied in the MMC case can also be applied here. For cell voltage balancing, the individual cell balancing or the sorting method can be implemented. Assuming the individual cell balancing method as detailed in [27] is implemented (opposed to sorting), considering all the control variables obtained above, the final modulation reference for the left and right arm is obtained as:

(26) $$\left\{\begin{array}{cc} v_{l}^{*} & =V_{DC}^{*}-\frac{v_{inv}^{*}}{2}+v_{Bal}^{*}+v_{Cell\_Bal}^{*}\\ v_{r}^{*} & =V_{DC}^{*}+\frac{v_{inv}^{*}}{2}+v_{Bal}^{*}+v_{Cell\_Bal}^{*} \end{array}\right.$$

where $V_{DC}^{*}$ and $v_{i}^{*}$ are converter modulation commands for each arm from the DC and AC controllers, respectively. The output as a result of (26) contains DC components, fundamental-frequency components (60 Hz), and switching-ripple components. To mitigate the ripple components, phase-shifted PWM is used for each chopper [21]. Figure 5 shows the general control system depicting important controllers structure.

## 4. Simulation Network and Result Discussion

### 4.1. Simulation Network

From (26), it is clear that the AC and DC references are decoupled from on another. In a grid feeding PV application, the physical MPPT boost stage can be excluded. In which case, the DC current controller in Figure 4c servers only as an emergency controller for DC FRT. Instead, $V_{DC}^{*}$ is modulated for MPPT based on the reference generated by the employed MPPT algorithm. In this article, this is demonstrated in simulation only as a means of further demonstrating the wide DC-bus operation.

In order to demonstrate the converter capability, three cases (general operation and DC FRT test, STATCOM operation and PV and grid interfacing) of a 1.8 kVA configuration with three sub-modules per arm of the topology as in Table 1 are simulated in the PLECS environment. For near practical simulation, the arm impedance is intentionally set different. For converter modulation, phase-shifted unipolar-Sinusoidal PWM with a carrier frequency of 10 kHz was implemented. From Equation (21) and Figure 3, the required minimum capacitance with ten percent ripple voltage (ΔV=0.1) for operation with a minimum of 45 V DC-bus is approximately 2.89 mF as described in Section 2.3. The general simulation procedures are as follows:

- The capacitor voltage is first charged via a pre-charge resistor.
- The converter is synchronised with the grid via PLL.
- After grid synchronization, the capacitor voltage is boosted to the rated 270 V.
- Power is then exchanged between the grid and DC-bus.

### 4.2. Simulation Result and Discussion

#### 4.2.1. Case I (General Operation and DC FRT Test)

As the name implies, this subsection investigates the converter general operation and DC FRT performance. For this, a variable DC source is connected to the DC-bus and 1.8 kW of power is then exchanged between the grid and the source (from 0.4 s: rectification, and from 1 s: inverter mode). Where negative current implies receiving, and positive, sending. During this, the DC-bus voltage is varied from 45 V to 100 V (at 1.5 s), and a DC-bus short circuit test is also carried out (from 1.8 s to 2 s).

**Figure 6:**
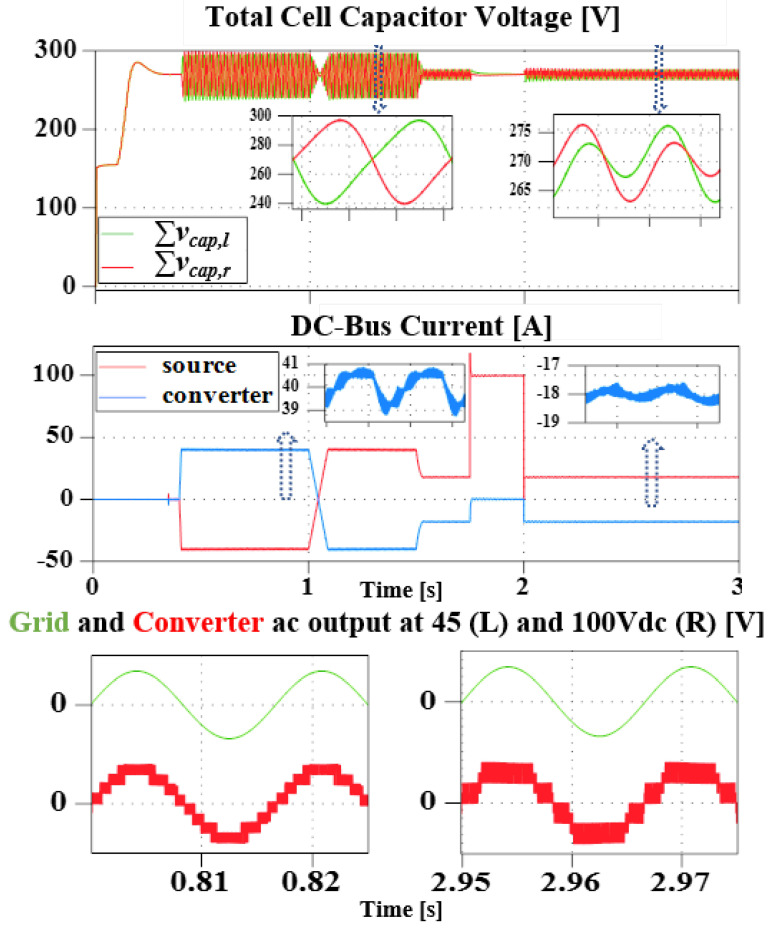
Simulation result showing the total left and right arm cell capacitors voltage, DC-side currents (both the source and the converter) during transients and steady-state, as well as the grid and converter AC output voltage during 45 V (**Left**) and 100 V (**Right**) DC-bus operation.

Figure 6 shows the converter total capacitors’ voltage, DC-side currents (source, and converter), as well as the grid and converter AC voltage (top to bottom, respectively). As seen, successful boosting to 270 V reference was achieved. Although with ripple during transients, it is within the calculated value and the converter returns to steady-state. Further more, in Figure 6, it is confirmed that during the fault period, FRT is achieved and the converter resumed operation once the fault is cleared. Furthermore, due to the action of the arm balancing control, sinusoidal ripple could be observed in the DC.

**Figure 7:**
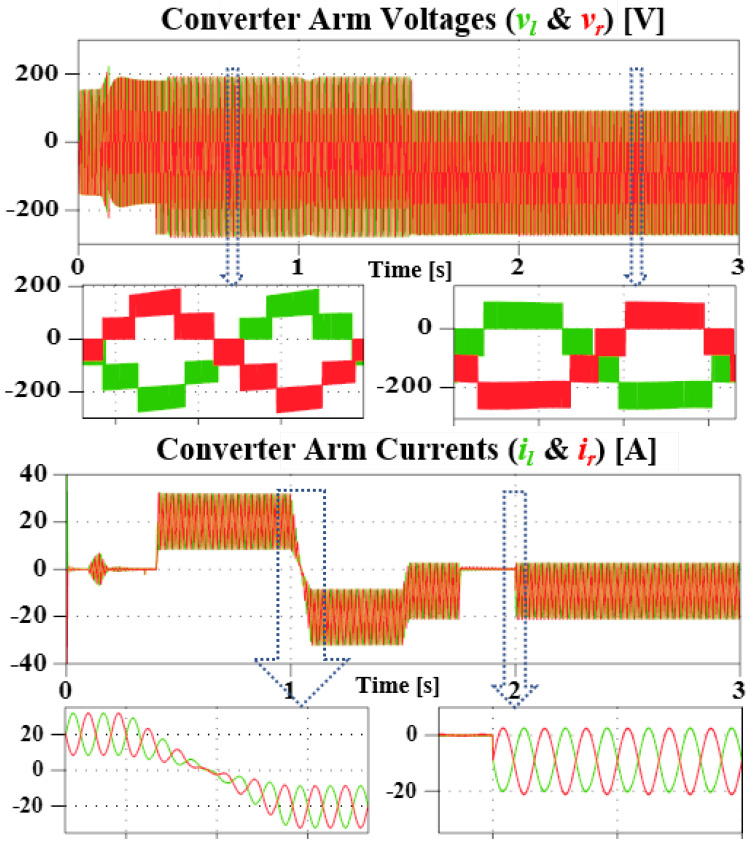
Simulation result of the converter arms output voltage, and output current during transients and steady-state.

The converter arm components during the simulation period is shown in Figure 7. It can be confirmed that both DC and AC components are present in the arms. Regardless of the difference in the arm impedance, the arms are approximately balanced and the converter does not lose stability even during the fault period. However, it is observed that an increasing DC-bus voltage reduces the inserted arm voltage level. Although the fundamental is correctly 180${}^{\circ }$ out of phase, the switching components slightly differ, and as a result, a distorted output AC waveform $v_{i}nv)$. Further studies will be required to fully resolve this.

#### 4.2.2. Case II (STATCOM Operation Test)

In case II, the converter is operated at 100 V DC and in rectification mode only. During 1 kW of active power transfer, approximately 0.8 kVar of reactive power exchange is requested at 1 s (ramped), then a DC SC fault is triggered at 1.5 s.

**Figure 8:**
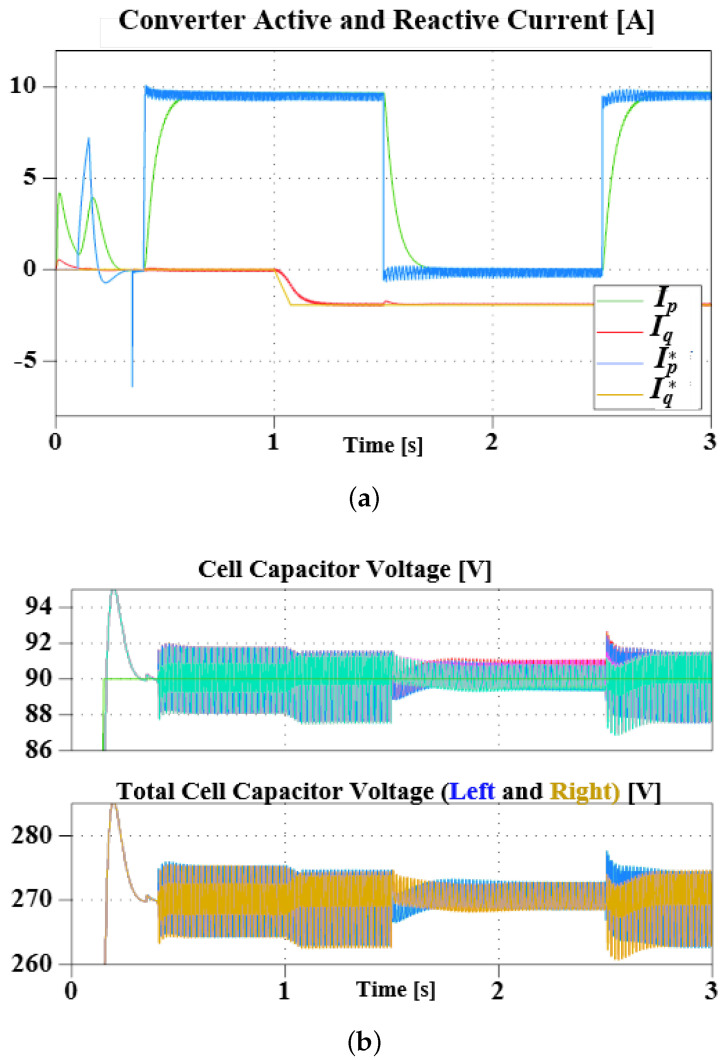
Simulation showing converter active and reactive current (**a**), and arm capacitors voltages during transients and steady state (**b**).

Figure 8 illustrates the converter response. As in Figure 8a, approximately zero active power is exchanged during the SC fault. However, during this period, the converter acts as a STATCOM by maintaining reactive power exchange with the grid. Likewise, the converter maintains the reference capacitor voltages, as in Figure 8b. Once the fault is cleared, the converter resumes power transfer to the source. The delayed transient of the currents ($I_{p}\;\mathrm{and}\;I_{q})$ is due to the bandwidth of the low-pass filters used in its calculation.

#### 4.2.3. Case III PV and Grid Interfacing

As aforementioned in Section 4.1, to further demonstrate the wide DC-bus operation, a grid feeding PV interfacing case is presented. In this case, the reference from the MPPT controller directly modulates $V_{DC}$ to achieve maximum power tracking. The PV units modeled in this simulation correspond to an array of three parallel-connected KC200GT solar modules, with 54 series-connected solar cells in each KC200GT module. The PV system was modeled as in [28], and a basic incremental conductance algorithm for MPPT, as described in [29], was implemented. Since the sole purpose is to demonstrate the wide DC-bus operation, optimization of the MPPT algorithm was not considered.

**Figure 9:**
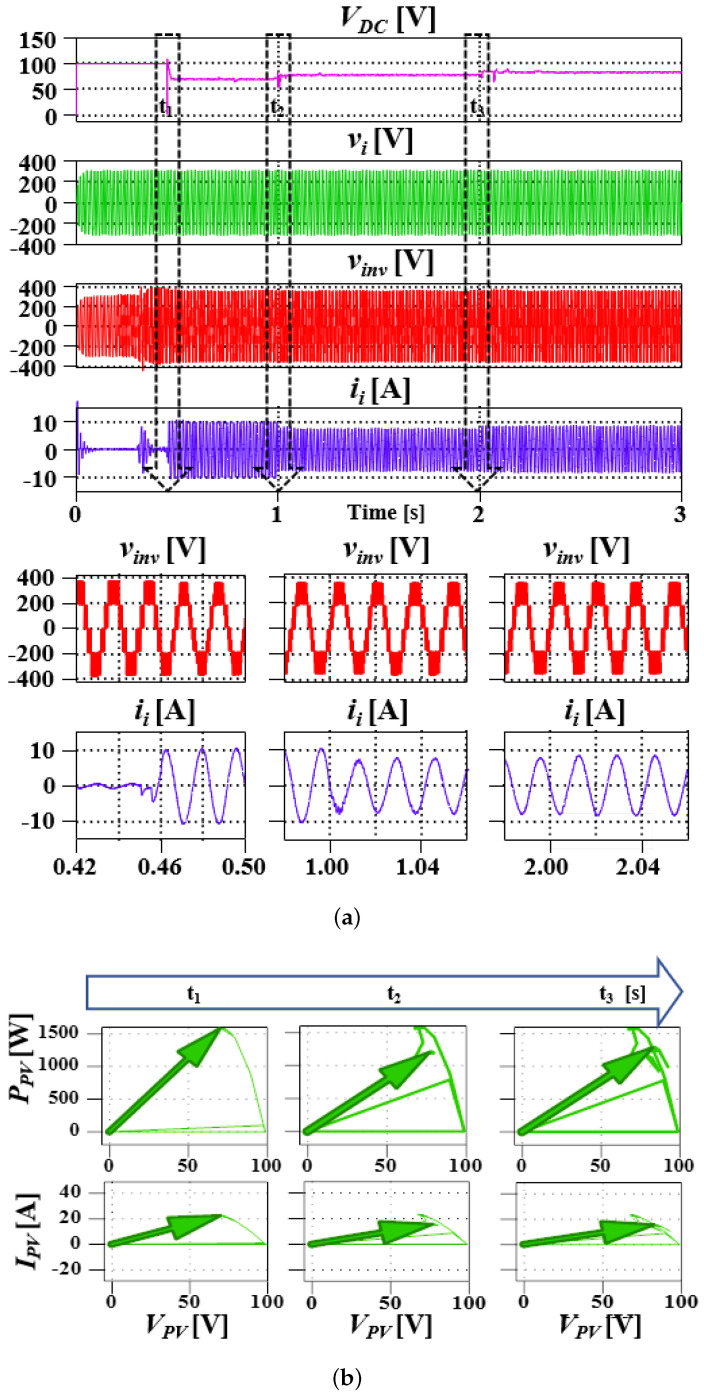
System’s DC (PV) grid voltage, current and converter AC output voltage in response to various change in PV conditions (**a**), and the PV P∼V and I∼V characteristics curve demonstrating the converters response to change in weather conditions (**b**).

**Figure 10:**
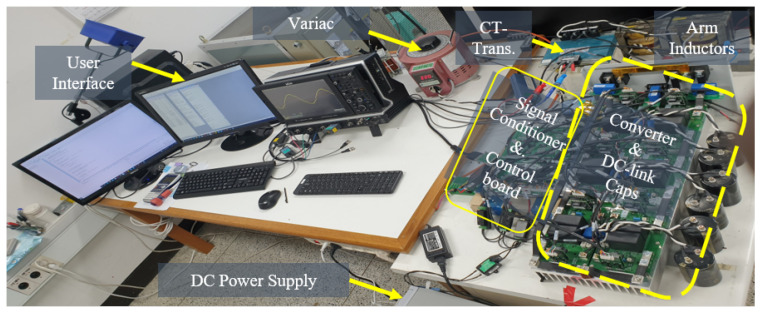
Experiment setup.

The PV module is connected to the converter at $t_{1}$. Through energy balance of the cells, power is dispatched to the grid. To evaluate the system performance, at $t_{2}\;65\%$ partial shading of one of the connected panels is simulated and, at $t_{3}$, the temperature varies from a nominal value of 25 °C to 10 °C and the converter response is as depicted in Figure 9, where $v_{inv}$ is the converter output voltage across the secondary side of the transformer. Figure 10 illustrates the converter response to the various test conditions in achieving MPPT. In each case, the converter is able to find a new maximum power point by following the reference from the MPPT algorithm. As can be verified by varying the converter’s DC component to produce optimal output for maximum power extraction as referenced by the MPPT algorithm, MPPT is achieved for each case.

## 5. Experimental Network and Result Discussion

### 5.1. Experiment Result and Discussion

With the exception of the minimum DC voltage being changed to 60 V, similar to the simulation section, for 1.8 kVA small-scale experimentation, the parameters are as shown in Table 2. This change is as a result of the current limitation of the DC power supply. A TI TMS320F28377S DSP was used for signal sampling and processing, control law implementation, and PWM generation. A combination of a digital to analogue (DA) converter and actual probes were used for waveform capture and both displayed on an oscilloscope. Data from actual probes and the DA are displayed with manually inscribed divisions. Instead of control suite, an easyDSP module was used for real-time command communication with the DSP. Although convenient for communication and testing, due to lengthy control code and communication speed limitation of the easyDSP module, the switching frequency is limited to 10 kHz. The converter and test-bed is as shown in Figure 10. It is a generic design for general purpose experimentation, hence not built to scale. A 150 A/1200 V SiCMOSFET was used for switching and the rest of the parameters is as depicted in Table 2.

#### 5.1.1. Phase-Locking

**Figure 11:**
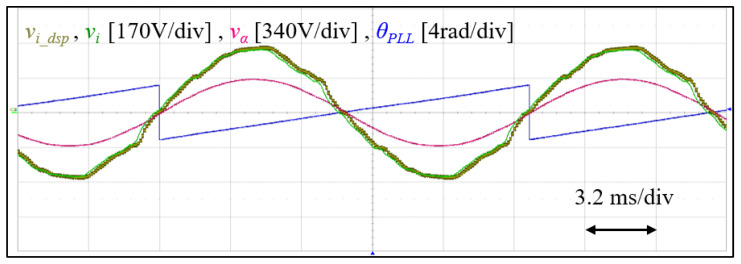
Experimental result showing Grid voltage and phase angle.

Figure 11 shows the grid voltage measured from an actual probe $v_{i}$, and from the DSP DA $v_{i\_dsp}$, alongside is the phase angle information and the stationary frame direct axis output of the *SOGI* block $v_{\alpha }$. As can be seen, the grid voltage exhibits harmonics, and slight propagation delay. However, $v_{\alpha }$ is harmonics free as expected.

#### 5.1.2. Converter Steady-State Operation Test

**Figure 12:**
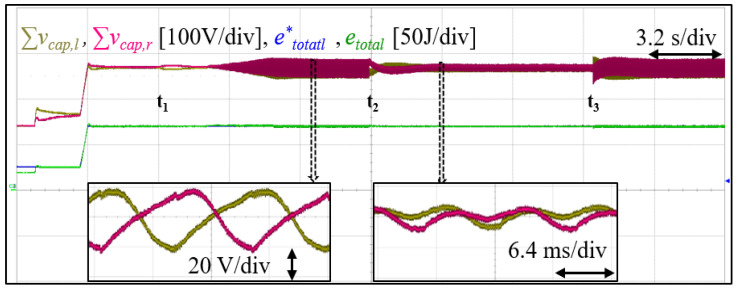
Experimental result showing converter total cell capacitors’ voltages, and total converter energy and its reference during transients and steady state.

**Figure 13:**
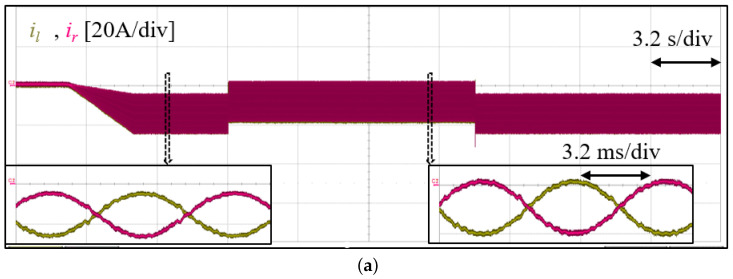

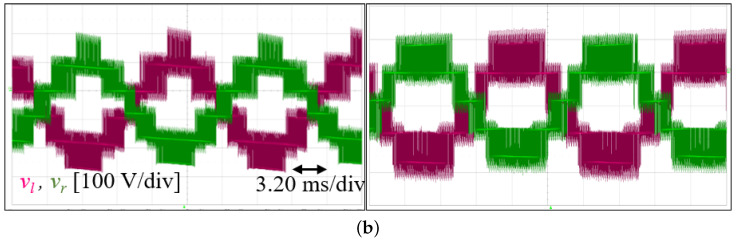
Experimental result of converter arms’ current (**a**), and voltage with: 60 V DC offset and 100 V DC offset (left and right, respectively) (**b**).

After pre-charging the cell capacitors and phase-locking with the grid, the cell voltage is boosted to required 270 V via the total energy controller. Thereafter, the DC power supply is connected to the converter at $t_{1}$. Next, 1.8 kW active power is transferred to the grid, during which the DC power supply voltage is stepped from 60 V to 100 V and back to 60 V ($t_{2}\;\mathrm{and}\;t_{3}$, respectively). Figure 12 shows the converter total arm capacitor voltages (left and right) and the total converter energy and its reference. As expected, the output energy tracks its reference and the arms total cell voltage remains balanced in both transient and steady state. Furthermore, it is verified that as the DC-bus voltage increases, the cell capacitor ripple becomes dominated by double the line frequency term, as visible in Figure 3. Figure 13 shows both arms current and voltage. Similar to the simulation result, there are both DC and AC components, and as expected, the arm current is well balanced. This validates the current relationship in (7) and as depicted in Figure 2a.

#### 5.1.3. STATCOM Operation Test and General stability

**Figure 14:**
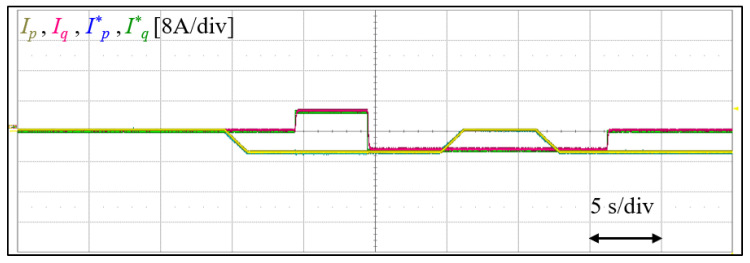
Experimental result showing the converter active and reactive components of the output AC $i_{i}$, and their references.

As in Figure 14, for STATCOM operation test, while sending 0.8 kW active power to the grid, 0.8 kVar of lagging and leading reactive power is exchanged, respectively. Then, the active component is gradually removed and restored in about 5 s. During this period, the converter remains stable and, as observed, the currents faithfully tracks their references.

**Figure 15:**
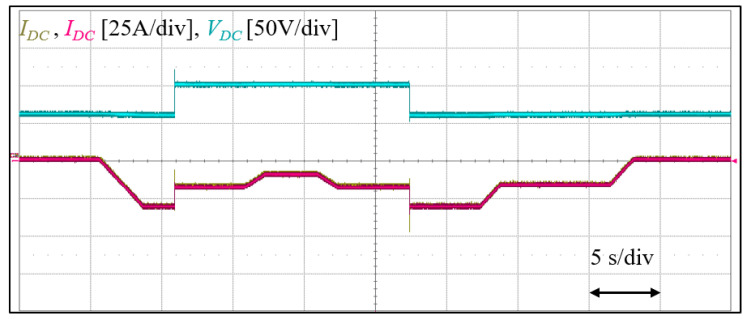
Experimental result of overall steady-state and transient stability test.

To further test the overall stability of the system, the converter DC current, and its reference along side the DC-bus voltage is observed while various active power test is carried out for both the minimum and maximum DC voltage. As in Figure 15, the converter responds as expected in tracking the DC reference even during the step change of the DC-bus voltage, while remaining stable. This further validates the control algorithm for wide DC-bus operation.

## 6. Conclusions

In this paper, a center-tapped transformer-based single-stage single-phase bidirectional multi-level converter was proposed for the improvement of wide DC-bus control performance and reliability. It was tested and verified via PLECS simulation and small-scale experimentation. The proposed topology achieved satisfactory results, as verified in the simulated and experimental results presented. It is found that a mismatch in the arm impedance can increase the burden on the arm balancing controller and result in DC ripple. Thus, it is essential to match the arm impedance to as close as possible. Furthermore, depending on the choice of total energy control, suitable arm balancing control should be adapted for effective and stable operation. The control of the AC is the same (although with slight differences) as conventional FB inverter(converter) control. This topology can be utilized in practical applications such as in BESS, PV, V2G, DG, and many more.

As verified through simulation, the open DC-bus operation with a PV source connected directly without a physical boost converter is theoretically possible. This can open pathways for connecting already existing large residential PV systems to the grid with better harmonic performance and possibly efficiency. Furthermore, it can improve the operation range of the system due to being able to operate with wider range of voltages, hence better power extraction in drastic weather conditions. However, further studies and experimental verification of this is still required, as well as comparative studies with current state of the art in terms of performance and economic viability.

With the continuous rise in transport electrification, rural areas are seeing an increase in mobility electrification from motorcycles, bicycles, vehicles, etc. However, this has not seen a corresponding rise in the availability of public chargers. This converter can find useful application in such areas for interfacing not only batteries to the grid, but also serve as a vehicle charger.

## Figures and Tables

**Table 1:** SYSTEM PARAMETERS.

Parameter	Value	Unit
Rated Output Power	1.8	kVA
Cells Per Arm ($N_{sm}$)	3	ea.
Arm Inductance	1	mH
${\mathrm{Arm}}_{l,r}$ Resistance	0.0012/0.001	Ω
DC Filter Capacitor Rating	100	μF
Cell Capacitor Rating	2.86	mF
DC-bus Voltage	45∼100	V
Grid RMS Voltage/Frequency	220/60	V/Hz
Switching Frequency	10	kHz
Transformer Turns Ratio	2:1	$N_{1}$:$N_{2}$

**Table 2:** SYSTEM PARAMETERS.

Parameter	Value	Unit
Rated Output Power	1.8	kVA
Cells Per Arm ($N_{sm}$)	3	ea.
Arm Inductance	1	mH
${\mathrm{Arm}}_{l,r}$ Resistance	Not Measured	Ω
DC Filter Capacitor Rating	100	μF
Cell Capacitor Rating	2.86	mF
DC-bus Voltage	60∼100	V
Grid RMS Voltage/Frequency	220/60	V/Hz
Switching Frequency	10	kHz
Transformer Rating	6.5	kVA
Transformer Turns Ratio	2:1	$N_{1}$:$N_{2}$
NF DC Power Supply	3	kW

## Data Availability

Not applicabl.
